# *Rhodnius prolixus* and *R. robustus* (Hemiptera: Reduviidae) nymphs show different locomotor patterns on an automated recording system

**DOI:** 10.1186/s13071-016-1482-9

**Published:** 2016-04-27

**Authors:** Márcio G. Pavan, Jessica Corrêa-Antônio, Alexandre A. Peixoto, Fernando A. Monteiro, Gustavo B. S. Rivas

**Affiliations:** Laboratório de Epidemiologia e Sistemática Molecular, Instituto Oswaldo Cruz, FIOCRUZ, Rio de Janeiro, Brazil; Laboratório de Biologia Molecular de Insetos, Instituto Oswaldo Cruz, FIOCRUZ, Rio de Janeiro, Brazil; Instituto Nacional de Ciência e Tecnologia em Entomologia Molecular (INCT-EM)/CNPq, Rio de Janeiro, Brazil; Present address: Laboratório de Bioquímica e Fisiologia de Insetos, Instituto Oswaldo Cruz, FIOCRUZ, Rio de Janeiro, Brazil

**Keywords:** *Rhodnius prolixus*, *Rhodnius robustus*, Triatominae, *Rhodnius*, Locomotor activity, Chronobiology, Behaviour, Cryptic species

## Abstract

**Background:**

Circadian rhythms of triatomines, vectors of the etiological agent *Trypanosoma cruzi* responsible for Chagas disease, have been extensively studied in adults of the two most epidemiologically relevant vector species, *Rhodnius prolixus* and *Triatoma infestans*. However, little attention has been dedicated to the activity patterns in earlier developmental stages, even though triatomine nymphs are equally capable of transmitting *T. cruzi* to humans. Because circadian rhythms may differ even between closely related species, studies that focus on this behavioral trait can also be used to shed light on the taxonomy of controversial taxa, which becomes especially relevant regarding vector species.

**Methods:**

We compared the daily locomotor activity patterns of second- and third-instar nymphs of *Rhodnius prolixus* and *Rhodnius robustus* in order to unveil possible behavioral differences between these cryptic species. Mitochondrial and nuclear markers were sequenced to confirm species identification.

**Results:**

Nymphs of both species had a bimodal pattern of locomotion and similar daily activity patterns, but *R. prolixus* is more active under light/dark cycles and depicts a more pronounced activity rhythm under constant darkness conditions.

**Conclusions:**

We describe the implementation of an often-used automated method for the recording of individual locomotor activity to differentiate sibling species of *Rhodnius* with distinct epidemiological relevance. The higher levels of activity observed in the nymphs of *R. prolixus* could potentially contribute to increased vector capacity.

**Electronic supplementary material:**

The online version of this article (doi:10.1186/s13071-016-1482-9) contains supplementary material, which is available to authorized users.

## Background

American trypanosomiasis (Chagas disease) is a neglected vector-borne disease caused by *Trypanosoma cruzi* (Kinetoplastida: Trypanosomatidae) and transmitted to humans mainly via infected feces of triatomine bugs (Hemiptera: Reduviidae). It is believed that 6–7 million people are estimated to be infected, mostly in Latin America and the Caribbean [1]. Disease prevention principally depends upon eliminating household-infesting triatomines since there is no vaccine or practical treatment on a large scale [2].

*Rhodnius prolixus* and *Rhodnius robustus* (*sensu lato*) (*s.l*.) are members of a cryptic species complex that includes at least six different lineages [3]. While *R. prolixus* is the main domestic Chagas disease vector in Colombia, Venezuela and certain areas of Central America [4, 5], *R. robustus* lineages are exclusively sylvatic and distributed throughout the Amazon region [3]. Although it is relatively well established that *R. prolixus* and *R. robustus *(*s.l*.) are *bona fide* species based on genetic markers [3, 6–8], no behavioral or morphological evidence has ever been produced to back up the molecular evidence.

The circadian control of insect activity rhythms [9, 10] is an area of behavioral science particularly relevant for the understanding of epidemiologically-related questions as it affects the time and the degree of contact between either the vector and the host or the vector and insecticide-sprayed surfaces [11, 12]. Moreover, behavioral studies conducted on sibling species might help identify evolutionary processes responsible for the formation of natural reproductive isolation barriers for gene exchange [13, 14].

Circadian rhythms of triatomines have been extensively studied in adults of the two most epidemiological relevant vector species, *R. prolixus* and *Triatoma infestans*. In both species these rhythms seem to control basic biological processes such as reproduction, foraging, breeding, oviposition [15, 16], dispersion [17] and host-seeking [18, 19]. Besides the light-dark and temperature cycles [20], the presence of a host seems to be an important *zeitgeber* [21]. As a foraging strategy, *R. prolixus* and *T. infestans* spend the day in refuges that are out of predator reach (in vertebrate nests and burrows in sylvatic habitats, or cracks and crevices when inside human dwellings) and become active at night when they leave to blood feed [22]. It is also known that *R. prolixus* males will also readily leave their shelters induced by the presence of females [23]. The nocturnal activity patterns displayed by adult triatomine bugs are generally bimodal. The first peak occurs just after dusk and corresponds to host-seeking activities, whereas the second is at dawn, interpreted as an effort by the insect to search for an appropriate daytime shelter [20, 22, 24].

Despite extensive studies on the locomotor activity patterns of adult *R. prolixus* and *T. infestans*, little attention has been dedicated to the activity patterns in earlier developmental stages even though triatomine nymphs are equally capable of transmitting *T. cruzi* to humans.

Locomotor activity rhythm is governed by molecular mechanisms, which in turn are regulated by a number of clock genes [25, 26]. Thus, interspecific differences in activity patterns should indicate evolutionary divergence [13]. Because circadian rhythms differ even between closely related species [13, 27], studying these rhythms may help to resolve controversial taxonomic issues. Therefore, the aim of the current work is to compare the daily locomotor activity patterns of second- and third-instar nymphs of *R. prolixus* and *R. robustus* in order to unveil possible behavioral differences between these cryptic species.

## Methods

### Species identification

We used in all assays laboratory-reared *R. prolixus* and *R. robustus* (*s.l*.) collected in Guatemala (Apr/2004) and Peru (June/1994), respectively. Since these are cryptic species, the identity and purity of colonies were confirmed through DNA sequence analyses and phylogenetic reconstructions. Twenty specimens of each colony were sequenced for two molecular markers: (i) a 264-bp fragment of the fourth intron of the single-copy nuclear Transmembrane protein 165 gene (TPS165), referred to as *AmpG*, that contains a SNP that differentiates *R. prolixus* from *R. robustus* (*s.l*.) [8] and (ii) a 682-bp fragment of the mitochondrial cytochrome *b* gene (*cytb*), which differentiates not only *R. prolixus* from *R. robustus * (*s.l*.), but also discriminates all four cryptic species of *R. robustus* complex (*R. robustus* I-IV) [3, 6].

DNA samples were extracted from 1 to 2 legs of each specimen using the Promega kit, according to the manufacturer’s recommendations. *cytb* and *AmpG* fragments were amplified as previously described [6, 8]. Purification of PCR products was carried out with the Hi Yield™ Gel/PCR DNA Extraction Kit (Real Genomics™). Forward and reverse strands were subjected to fluorescent dye-terminator cycle sequencing reactions (ABI Prism® BigDye® Terminator v3.1 Cycle Sequencing Kit, Applied Biosystems) and run on an ABI 3730 automated sequencer.

Bayesian phylogenetic trees were reconstructed according to Abad-Franch et al. [3] and Pavan et al. [8] (see Additional file 1 for detailed specifications).

### Locomotor activity recording

Second- and third-instar nymphs of *R. prolixus* and *R. robustus *(*s.l*.) fed on chicken blood ten days prior to experimentation. Only nymphs not fed to repletion were selected, and specimens that moulted until the date of the experiment were discarded. Insects were individually transferred to 1 × 7 cm glass tubes with both ends properly sealed with Parafilm® M (Sigma-Aldrich). Locomotor activity analyses were performed with a larger version of the *Drosophila DAM5 Activity Monitoring System* (Trikinetics Inc., Waltham, MA, USA), as previously described [13, 28]. This system incorporates an infrared interruption method that detects movement every time the insect crosses the beam. Each movement detected by the monitor was recorded with computer software (*DAMSystem3 Software*).

All monitors were placed in a Precision Scientific Incubator (Model 818), under a relative humidity of 75 % at constant temperature of 25 °C. Activities were recorded under an artificial photoperiodic regime of 12:12 LD (cycles of 12-h of light and 12-h of darkness) for five days, followed by 23 days of 24-h DD (constant darkness) and five days of 12:12 LD. The experimental procedure was performed in triplicate. These data were pooled as a preliminary statistical analysis and showed no significant differences between the repetitions.

Graph comparison of daily activity patterns between *R. prolixus* and *R. robustus* in 12:12 LD was performed in Excel™ (Microsoft^©^), depicting the average activity of five days under this regime. Weighted moving averages of data were used to “smooth” data variation. Only insects with noticeable activity lasting for at least six consecutive days were analyzed. For these triatomines, the total activity under 12:12 LD was estimated by summing the locomotion recorded during five consecutive days. In parallel, we also calculated the proportion of nocturnal activity, dividing the sum of activity recorded during the dark phase by the total amount of daily activity, as previously described [13]. Proportions were normalized by the arcsine square root method in order to be further analyzed with parametric statistics. Comparisons were conducted based on the Analysis of variance (ANOVA).

After five days under 12:12 LD, all nymphs were submitted to 23 days of constant dark conditions (DD). Under this regime, triatomines normally depict a free-running pattern of locomotor activity which represents an apparent persistence of the endogenous rhythm and may differ from the 24-h period. To determine this pattern, all individual actograms were smoothed (smoothing Gaussian standard deviation tool in software ActogramJ) to reduce the noise observed in the periodograms [29]. Then, the free-running period length (τ) of each insect was calculated with the *χ*^2^ periodogram algorithm [30] in ActogramJ [29]. The free-running period length was calculated in three different time windows: (i) considering the first ten days (τ_1–10_) which was pointed out by Refinetti [31] as being optimal; (ii) the last thirteen days (τ_11–23_) which depicts any uncoupling effect of oscillators due to long starvation period; and (iii) all 23 days under DD (τ_23_), aiming to define if longer days in DD could increase the resolution of the analysis.

We estimated the strength (power) of the rhythm which is defined as the amplitude from the top of the peak to the confidence level in the *χ*^2^ periodogram [32]. Liu et al. [32] previously showed that some *per* mutants of *Drosophila* are arrhythmic, and therefore, their “power” is zero. Also, other minor impairments on *per* expression into the brain of *Drosophila* specimens could affect the strength of rhythm, which could be visualized by lower power levels compared to wild-type flies [32]. More recently, this method has been adopted to evaluate the effect that the knockdown of clock genes has on the rhythm of non-*Drosophila* species [33–35]. Therefore, estimates of power levels are effective for evaluating the rhythmicity of such organisms and can be useful to depict possible differences among *Rhodnius* species under laboratory conditions.

### Actogram plotting and acrophase calculation

Only insects that demonstrated continuous activity throughout all the experiments and a *χ*^2^ periodogram with a peak above the confidence level were selected to represent average actograms of *R. robustus*(*s.l*.) and *R. prolixus*. From the average actogram, we calculated the acrophase (with the acrophase tool in ActogramJ), a parameter that indicates the central phase of the activity cycle which generally coincides with the main peak of activity [36].

### Statistical analysis

Statistical analysis and graphs were accomplished with the GraphPad Prism 5 package (Prism, La Jolla, CA), as previously described [34, 37]. We checked if all parameters analyzed followed a Gaussian distribution with the Shapiro-Wilk normality test (*P* < 0.05). Only data from the 23-day free-running treatment under DD (τ_23_) passed this test and thus, were submitted to the parametric unpaired Student’s *t* test (with 5 % significance level). The other parameters (τ_1–10,_ τ_11–23_ and “power” level) were analyzed with the Mann-Whitney test (*P* < 0.05). The proportion of nocturnal activity and the total daily activity were statistically compared between the two species. One-way ANOVA was performed for this comparison in the R environment [38].

## Results

### Species identification

**Fig. 1:**
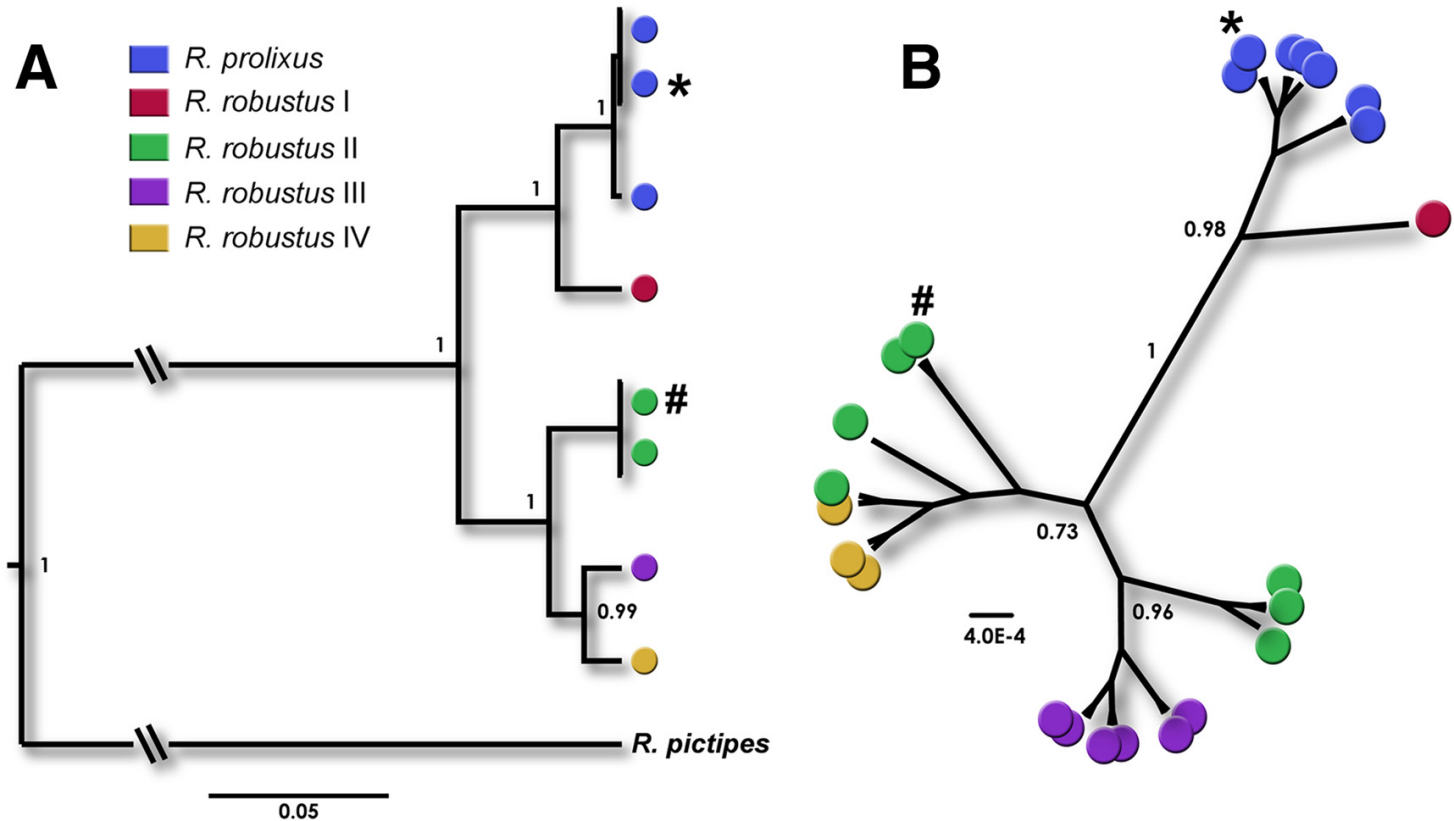
Phylogenetic analyses of *R. prolixus* and *R. robustus* (*s.l*.) Bayesian consensus trees of 9 *cytb* (**a**) and 24 *AmpG* sequences (**b**). Branches that connect *R. prolixus* + *R. robustus* and *R. pictipes* were reduced for a better visualization. Posterior probabilities > 0.7 are shown for key nodes. Node circles represent sequences of *R. prolixus* and *R. robustus * (*s.l*.) and are color-coded (see color key). The position of *R. prolixus* and *R. robustus* II sequences were highlighted with symbols “*” and “#”, respectively

Phylogenetic analyses confirmed the identification of the specimens used as *R. prolixus* and *R. robustus* II (*sensu* Pavan et al. [8]). The 20 *R. prolixus* specimens had the same mitochondrial *cytb* haplotype and nuclear *AmpG* genotype as EF011726 and JQ432888, specimens collected in Venezuela and Guatemala, respectively [7, 8]. The other 20 specimens analyzed had the same haplotype and genotype of *R. robustus* II from Napo, Ecuador (AF421341 and JQ432896, respectively) [7, 8] (Fig. 1).

### Locomotor activity pattern

**Fig. 2:**
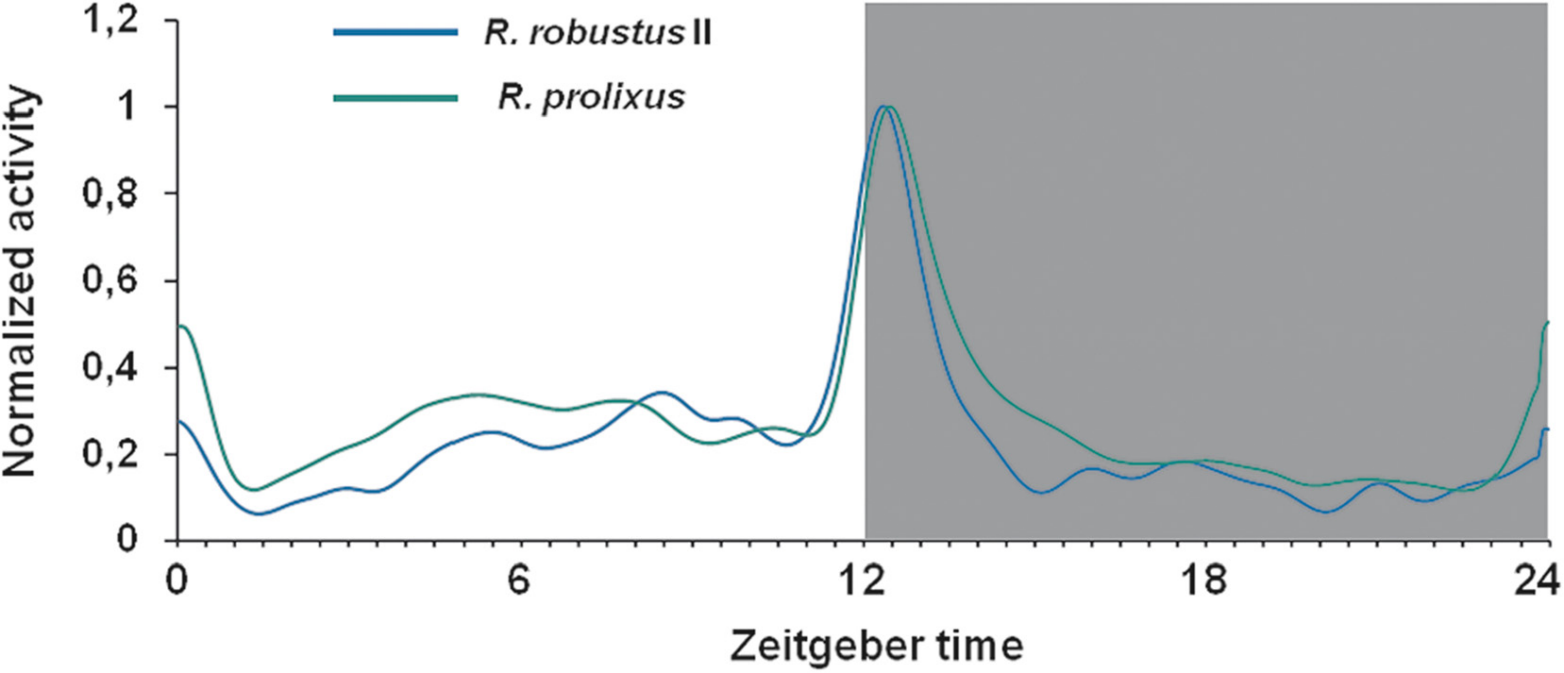
Daily activity profile of *R. robustus* II and *R. prolixus* under 12:12 LD. Average locomotor activity of *R. robustus* II (*blue lines*, *N* = 30) and *R. prolixus* (*green lines*, *N* = 65), depicted by average values of five days of recording. The horizontal white bar represents the light phase and the black bar indicates the dark phase. ZT = *Zeitgeber* Time within a light/dark cycle experiment. ZT 0 corresponds to the lights-on event and ZT 12 to the lights-off event

When submitted to light/dark cycles (12:12 LD, 1000 lux), nymphs of *R. robustus* II and *R. prolixus* showed similar activity patterns throughout the day and shared the same circadian pattern (Fig. 2). At dusk, individuals of both species increased their activity one hour before lights on/off transition, leading to a peak at early night. Then, locomotion starts to decrease until basal levels throughout the night. The highest levels of activity were recorded during this interval (ZT11-ZT14) and, together with night activity, represent the major part of daily activity in both species (58.2 % *R. robustus* and 53.9 % for *R. prolixus*). One-way ANOVA comparing the proportion of activity during scotophase revealed that there was no difference between species (*F*_(1,135)_ = 0.38; *P* = 0.538). Also, we observed a minor peak at morning followed by a discrete activity during daytime.

**Fig. 3:**
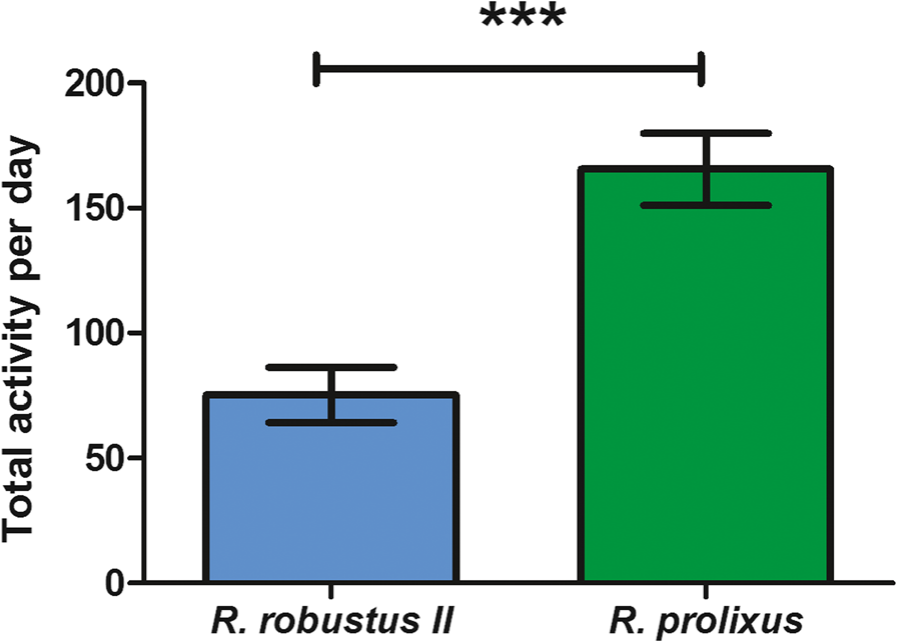
Locomotor activity levels of *R. robustus* II and *R. prolixus* under 12:12 LD. Total locomotor activity per day of *R. robustus* II (*blue bar*, *N* = 30) and *R. prolixus* (*green bar*, *N* = 65), calculated with five days of recording. Mann Whitney *t* test (***) *P* < 0.001

The main difference between species in the 12:12 LD condition concerned the total amount of activity, with *R. prolixus* being noticeably more active than *R. robustus* II (one-way ANOVA *F*_(1,135)_ = 19.90; *P* < 0.001; Fig. 3).

### Locomotor activity under constant dark cycles

**Fig. 4:**
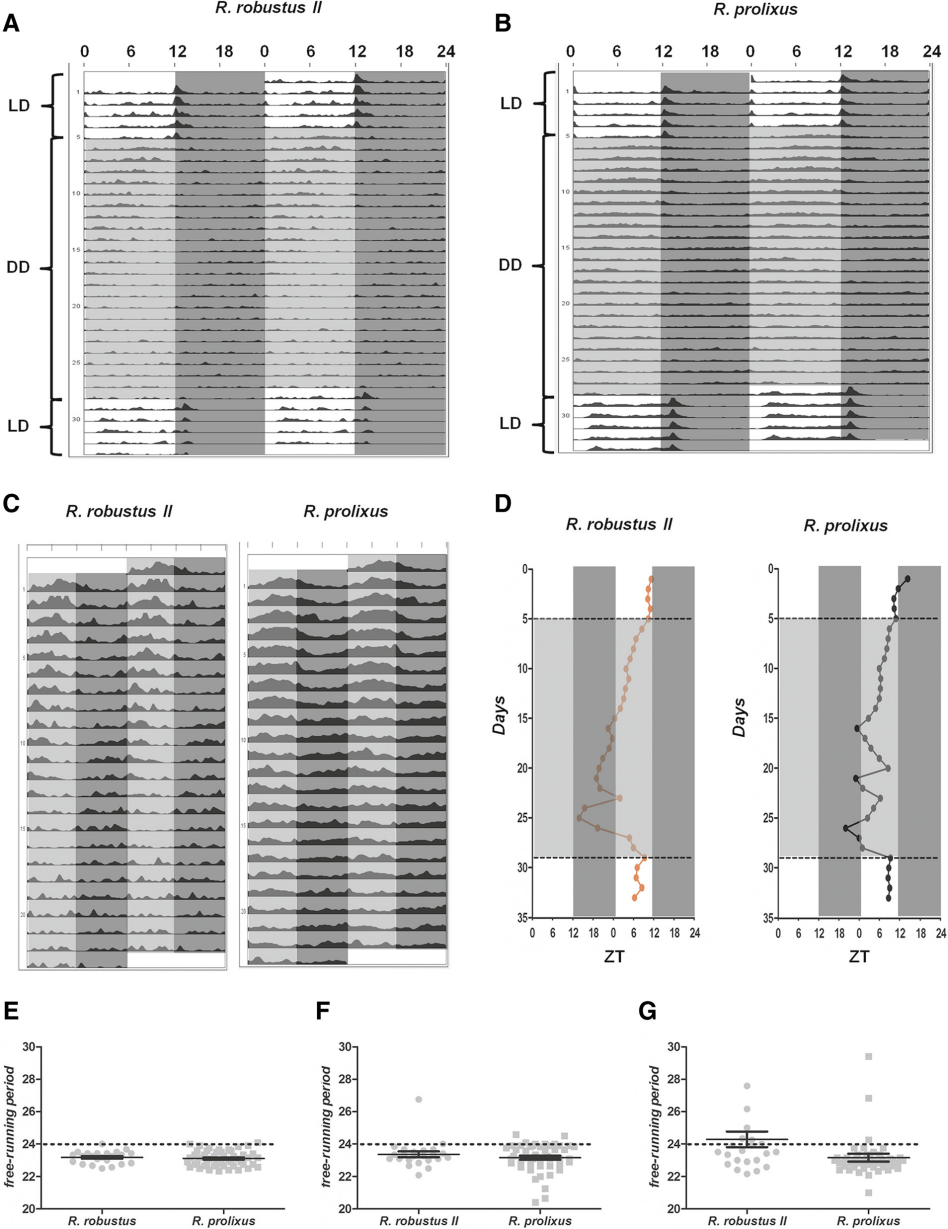
Locomotor activity/rest behavior of *R. robustus* II and *R. prolixus* under 12:12 LD followed by constant darkness (DD). Double-plotted actograms for *R. robustus* II (**a**; *N* = 23) and *R. prolixus* (**b**; *N* = 50) depicting five consecutive days under 12:12 LD followed by 23 days under constant darkness and reverting to another five days under 12:12 LD. **c** Smoothed double-plotted actograms of both species under constant darkness. The shaded dark grey areas indicate dark phase and light grey areas indicate the subjective days in constant darkness. **d** Acrophase of *R. robustus* II (red dotted lines) and *R. prolixus* (black dotted lines) during the 33 days of experiment. Dashed lines represent the transition from LD to DD (top) and from DD to LD (bottom). **e**-**g** Free running period length of *R. robustus* II (circles) and *R. prolixus* (squares), based on the whole 23-day period under DD (**e**), the first 10 days (**f**) and the last 13 days (**g**). Solid lines represent the average ± SEM. Dotted lines mark the 24-h period length

*R. prolixus* and *R. robustus* II specimens were also submitted to a regime of complete darkness (DD) for 23 days followed by an additional five days of 12:12 LD at 1000 lux. Figures 4a and b reveal that the overall activity of both species in darkness is much lower than the previous activity in 12:12 LD, being barely visible in actograms. Although reduced, the locomotor activity under DD is rhythmic in both species and presents a circadian period shorter than 24 h (Fig. 4c and Table 1).

**Table 1:** Locomotor activity rhythms of *R. robustus* II *and R. prolixus* in constant darkness

		Species		Mann-Whitney test	
		*R. robustus* II	*R. prolixus*	*U*	*P*
N		33	64	–	–
Proportion of rhythmic insects (%)	Total	69.70	78.13	–	–
	SR^a^	21.21	46.88	–	–
	WR^a^	48.48	31.25	–	–
Free-running period length (Mean ± SD)^b^	τ_23_	23.17 ± 0.07	23.10 ± 0.06	NS	NS
	τ_1–10_	23.37 ± 0.17	23.15 ± 0.12	NS	NS
	τ_11–23_	24.29 ± 0.48	23.16 ± 0.24	347.5	0.0069
Power levels (Mean ± SD)^c^	Power _23_	606.4 ± 71.96	799.0 ± 59.53	381.5	0.0219
	Power _1–10_	329.5 ± 37.19	472.3 ± 41.27	379.5	0.0206
	Power _11–23_	330.7 ± 34.97	398.7 ± 29.04	NS	NS

^a^SR-“Strongly rhythmic”. Proportion of insects (%) whose power levels were above 500. WR-“Weakly rhythmic”. Proportion of insects (%) whose power levels were below 500. See results for details

^b^Free-running period length (in hours ± SEM) was calculated in three different time windows: (i) considering all 23 days under DD (τ_23_); (ii) the first ten days (τ_1–10_); and (iii) the last thirteen days (τ_11–23_). See Methods for details

^c^The power of the rhythm was defined as the amplitude from the peak to the cutoff line (α = 0.05) in the *χ* ^2^ periodogram (Liu et al. [32]). See methods for details. The power level was calculated in three different time windows. Power _23_: considering all 23 days under DD. Power _1–10_: the first ten days. Power _11–23_: the last thirteen days

*NS,-* non-significant

Figure 4d displays a pattern of phase advance during the first 10 days. In the following days, the acrophase of both species started to be inconsistent, exhibiting phase shifts. These inconsistencies seem to appear earlier in *R. prolixus* than in *R. robustus*. This pattern of free running (especially into the last days in DD) strongly suggests that oscillators (i.e. endogenous clocks) have become decoupled in both species. Therefore, we calculated the free-running period based on three time-frames: the whole 23 day period under DD (Fig. 4e), the first 10 days (Fig. 4f) and also the last 13 days (Fig. 4g). Only the calculations of the last 13 days resulted in significant differences in the free-running period between species (Fig. 4f and Table 1). Although this observation could be interpreted as a further evidence of interspecific difference, we feel that it could also have resulted from the decoupling oscillators.

Although free-running periods were similar in both species, more *R. prolixus* specimens were rhythmic and presented higher power levels than *R. robustus* II (Table 1). We observed, by visual inspection of individual periodograms, that some insects depicted a clear free-running pattern under constant darkness. In these cases, the power levels were always above 500 (see Additional file 2). On the other hand, other rhythmic triatomines presented a more complex free-running pattern and power levels below 500 (Additional file 2). Therefore, we decided to classify them into two groups defined as “strongly rhythmic” and “weakly rhythmic”. The power levels in Table 1 clearly display a larger number of strongly rhythmic *R. prolixus* specimens. This demonstrates that *R. prolixus* has a more evident behavioral rhythm than *R. robustus* II under constant conditions.

## Discussion

This paper compares the daily activity rhythms between nymphs of the sibling Chagas disease vector species *R. prolixus* and *R. robustus* II, under laboratory conditions. The most salient features observed were the higher activity levels and more pronounced rhythmicity of *R. prolixus*. Although earlier results based on mitochondrial and nuclear markers strongly indicate that *R. prolixus* and *R. robustus* II are valid species [3, 6–8], here we present the first behavioral evidence in support for their separation based upon molecularly certified insects.

Previous behavioral analyses in triatomines have successfully used ingenious systems to record locomotor activity (e.g. [20, 39]). Herein, we evaluated the individual circadian patterns of two *Rhodnius* species with a commercial automated recording system of locomotor activity widely used in insect chronobiology studies [13, 27, 40–42]. Automated systems are better suited for the collection and compilation of behavioral data since activity is not recorded by visual inspection, therefore potentially minimizing human error.

Behavioral analyses under artificial conditions are more suitable to unveil species-specific patterns that are driven by the endogenous clock [13] since insect activity under natural conditions is influenced by multiple factors, such as social and host cues as well as environmental oscillations. Our results depicted the patterns of daily activity expected for both *Rhodnius* spp. , with two major peaks of activity: one at dawn, most likely associated with the search for refuge, and the other at dusk, related to host-seeking behavior [20, 22]. Besides main peaks, we also observed in both species a basal activity, irrespective of light presence. Although fifth-instar nymphs and adult triatomines seem to have a negative phototactic response [22], *Rhodnius* second- and third-instar nymphs did not show reduced activity during the light phase.

The daytime activity recorded here varies among bugs, with some presenting small and sparse bouts of activity and others with continuous and high mobility levels. This activity is clearly clock-controlled, because it persisted visibly in constant darkness for more than one week and free-ran with a period of less than 24 h (Fig. 4c). In any case, it seems that the daytime inactivity in *Rhodnius* nymphs is fragmented and discontinued. Other insects such as *Apis mellifera* and *Drosophila* also alternate states of deep sleep and wakefulness during the day [43, 44]. In the case of the former, sleep behavior has an ontogenetic dependence. Foragers (old bees) exhibit a nighttime consolidated sleep [45, 46], while young bees distribute their sleep throughout the day [47, 48]. We cannot discard the hypothesis that light avoidance in nymphs of *Rhodnius* is influenced by an ontogenetic effect, with younger nymphs being less sensitive to light than older nymphs and adults. Future experiments should help clarify this particular issue.

The results of power levels of activity in constant darkness clearly showed that *R. prolixus* has a circadian locomotor pattern more pronounced than *R. robustus* II. Moreover, nymphs of *R. prolixus* were more rhythmic than nymphs of *R. robustus* II, being able to sustain similar period lengths along the 23-day complete dark condition (Table 1). We suggest that these species have circadian pacemaker differences (i.e. different regulation of gene circadian expression and/or protein abundance). It is well known that circadian clock and metabolism are interconnected in many insect species [49]. Recent RNA interference work based on the knockdown of the clock gene *timeless* in *Aedes aegypti* and *Gryllus bimaculatus* showed that the reduction of *tim* expression impairs the circadian activity. This impairment weakens the behavioral response of the endogenous clock, which is marked by lower power levels in specimens injected with ds*tim* RNA [34, 50]. In addition, blood-feeding reduces circadian expression of *period*, *timeless* and *Clock*, which is followed by lower levels of locomotor activity in *Aedes aegypti*, *Aedes albopictus,* and *Lutzomyia longipalpis* [28, 51]. Therefore, we suggest that the higher activity under LD and strong rhythmicity of *R. prolixus* might be a result of the circadian clock and metabolism acting together. In this perspective, we can hypothesize that *R. prolixus* digests blood faster than *R. robustus* II, as previously shown for morphologically identified *R. prolixus* and *R. robustus* (*s.l*.) from Venezuela [52, 53]. Hence, *R. prolixus* would search for blood meals more frequently, increasing the risk of transmission of *T. cruzi*.

This is the first study to show behavioral differences between genetically certified *R. prolixus* and *R. robustus* II, two sibling triatomine species of clearly distinct epidemiological relevance. In this respect, we speculate if behavioral differences between these two species could have an impact on their vectorial capacities. Recent work on mosquitoes might contribute for a better understanding of this idea. For instance, it was observed that two cryptic species of *Anopheles gambiae* exhibit similar patterns of flight activity, but one form is more active than the other [27]. The authors suggest that the different levels of activity between these species could affect host-seeking dispersal. Also, a previous work incorporating mathematical modeling indicated a positive correlation between locomotor activity and biting rate in *Aedes aegypti* [54]. Altogether, we believe that higher levels of activity in *R. prolixus* could contribute to an increase in its dispersal capabilities and blood feeding efficiency thus rendering it a more effective vector.

## Conclusions

We describe the implementation of an often-used automated method for the recording of individual locomotor activity to differentiate sibling *Rhodnius* spp. with distinct epidemiological relevance. The higher levels of activity and rhythmicity observed in *R. prolixus* nymphs could potentially contribute to increased vector capacity.

### Ethical approval

The animals used to maintain the insects at FIOCRUZ were treated according to the Ethical Principles in Animal Experimentation approved by the Ethics Committee in Animal Experimentation (CEUA/FIOCRUZ) approved under the protocol numbers P-54/10-4/LW12/11 and L-0061/08. Both protocols are from CONCEA/MCT (http://www.cobea.org.br), which is associated with the American Association for Animal Science (AAAS), Federation of European Laboratory Animal Science Associations (FELASA), International Council for Animal Science (ICLAS) and Association for Assessment and Accreditation of Laboratory Animal Care International (AAALAC).

## Additional files

Additional file 1: Protocol S1. Detailed methods used in phylogenetic reconstructions of *Rhodnius prolixus* and *R. robustus*. (DOCX 29 kb) Additional file 2: Individual actograms with different free-running patterns under constant darkness. A) Double-plotted actogram of a single nymph with a free-running pattern less than 24 h (left), which is easily observed by visual inspection and confirmed by *χ* ^2^ periodogram (right). The periodogram has a peak at 1405 min (~23 h) and the *P* value level for the actogram is displayed as a red line. The “power” value is defined as the amplitude of the peak, and is measured from the confidence level to the top. B) Double-plotted actogram of a single nymph, displaying a more difficult pattern to track by visual inspection (left). The *χ* ^2^ periodogram (right) has a peak of 1420 min (approximately 23 h). However, the graph is quite inconsistent and power level is considerably lower than depicted in A. Light and dark grey areas indicate the subjective day and night, respectively, in constant darkness. (TIF 733 kb)
