# Supplementary material for: Rhodnius prolixus and R. robustus (Hemiptera: Reduviidae) nymphs show different locomotor patterns on an automated recording system
Source: Parasit Vectors. 2016 Apr 27;9:239. doi: 10.1186/s13071-016-1482-9 (PMC4848847; doi:10.1186/s13071-016-1482-9)
Supplement: Additional file 1: — Protocol S1. Detailed methods used in phylogenetic reconstructions of Rhodnius prolixus and R. robustus. (DOCX 29 kb) [file 13071_2016_1482_MOESM1_ESM.docx]

**Additional file 1: Protocol S1. Detailed methods used in phylogenetic reconstructions of *Rhodnius prolixus* and *R. robustus*.**

*Bayesian phylogenetic reconstructions*

In summary, we used the jModeltest2 [50] to elect the Hasegawa, Kishino and Yano model with a proportion of invariable sites and four gamma parameters (HKY+I+G), as well as the Jukes-Cantor (JC) as the best-fit models of nucleotide substitution (based on the Bayesian Information Criterion) for the phylogenetic reconstructions for *cytb* and *AmpG*, respectively. Orthologous sequences from *R. prolixus* and other members of the *R. robustus* species complex (*R. robustus* I-IV) were added to the analyses (GenBank accession numbers AF421340, AF421341, AF421342, EF011723, EF011726, EF011727, JQ432866, JQ432870, JQ432880, JQ432888, JQ432891, JQ432893, JQ432895, JQ432896, JQ432906, JQ432915, JQ432968, JQ432970, JQ432974, JQ432976, JQ432983, JQ432985, JQ432988, JQ432991, JQ432993, JQ432994, JQ432996, JQ432997). One sequence of *Rhodnius pictipes* (GenBank accession number JX273157) was selected to root the *cytb* phylogram at the midpoint. A Bayesian phylogenetic tree for each data set was inferred in BEAST v1.8 [51], imposing the Yule process of speciation for all tree reconstructions. Three independent runs were performed for 5 x 10^7^ generations, with a 10% burn-in. Proper mixing of chains and convergence of parameters were confirmed by calculating the effective sample size (ESS) in TRACER 1.6 [52], excluding the initial 10% (burn-in) of each run. All estimated parameters had ESS > 10^4^. Runs were combined with LogCombiner and a maximum credibility tree based on the 10,000 trees (burn-in = 2,000) was generated for each marker with a posterior probability limit of 0.6 with Tree Annotator (both part of the BEAST package). Statistical support for clades was assessed by the posterior probability method.
