# Supplementary figures and images for: Rhodnius prolixus and R. robustus (Hemiptera: Reduviidae) nymphs show different locomotor patterns on an automated recording system
Source: Parasit Vectors. 2016 Apr 27;9:239. doi: 10.1186/s13071-016-1482-9 (PMC4848847; doi:10.1186/s13071-016-1482-9)

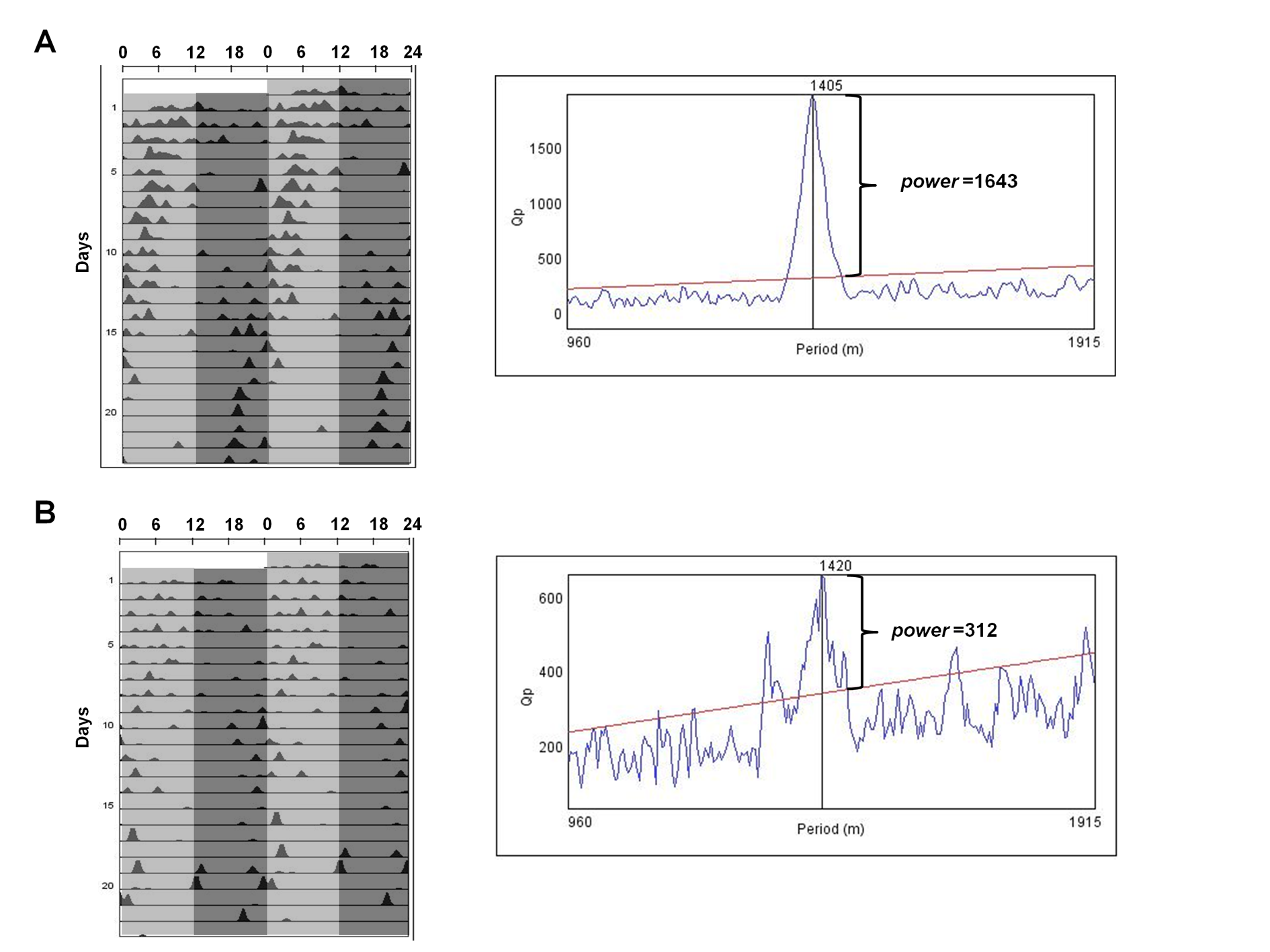

Supplement: Additional file 2: — Individual actograms with different free-running patterns under constant darkness. A) Double-plotted actogram of a single nymph with a free-running pattern less than 24 h (left), which is easily observed by visual inspection and confirmed by χ 2 periodogram (right). The periodogram has a peak at 1405 min (~23 h) and the P value level for the actogram is displayed as a red line. The “power” value is defined as the amplitude of the peak, and is measured from the confidence level to the top. B) Double-plotted actogram of a single nymph, displaying a more difficult pattern to track by visual inspection (left). The χ 2 periodogram (right) has a peak of 1420 min (approximately 23 h). However, the graph is quite inconsistent and power level is considerably lower than depicted in A. Light and dark grey areas indicate the subjective day and night, respectively, in constant darkness. (TIF 733 kb) [file 13071_2016_1482_MOESM2_ESM.tif]
